# George P. Rowell & Co.’s Book for Advertisers

**Published:** 1891-07

**Authors:** 


### GEORGE P. ROWELL & CO.'S BOOK FOR ADVERTISERS.

Geo. P. Rowell & Co., of New York, publishers of the American Newspaper Directory and of *Printers' Ink*, a journal for advertisers—the oldest and best known of all the advertising agencies—conduct their business in such a way as to make it a material benefit to both advertiser and newspaper publisher.

They furnish plans for an advertiser and prepare his advertisment. For their services—designing his advertisement and preparing his estimate—they make a sufficient charge to pay for the required service of persons competent to do the work well. They tell the advertiser what papers he should use and what the price will be. If the advertiser wishes them to place the advertisement in the papers, they do as he directs, and for that service the newspapers pay them. If the advertiser wishes to place his advertising through some other advertising agency, or to contract with the publishers, he is at liberty to do so, and the estimate furnished by Messrs. Rowell & Co. serves as a guide. It tells him where he is securing a bargain and where he is paying more than he ought.

Every one who is in need of information on the subject of advertising will do well to obtain a copy of Geo. P. Rowell & Co.'s *Book for Advertisers*, 368 pages, price one dollar. It is mailed, postage paid, on receipt of price, and contains a careful compilation from the American Newspaper Directory of all the best papers in the United States and Canada. It gives the circulation rating of every one and a good deal of information about rates and other matters pertaining to the business of advertising.

Whoever has made himself acquainted with what may be learned from this book will admit that from its pages one may gather pretty much all the information that is need to perfect an intelligent plan of advertising. It is not a complete newspaper directory. It is much better; for although it names barely one-third of the newspapers published, it does enumerate every one of the best, and all that a general advertiser is likely to have occasion to use.

Among the papers named in it THE HOMŒOPATHIC PHYSICIAN occupies the position to which its merits entitle it.

